# C4orf3 Regulates HIF-1α Degradation Under Hypoxic Conditions and Contributes to the Malignant Phenotype in Small Cell Lung Cancer

**DOI:** 10.7150/jca.127942

**Published:** 2026-01-23

**Authors:** Keita Sakanashi, Hideya Onishi, Naoya Iwamoto, Yoshiyuki Nakanishi, Shinsaku Itoyama, Shogo Masuda, Keigo Ozono, Kosuke Yanai, Katsuya Nakamura, Masayo Nagami, Kenichi Nishiyama, Masayuki Kojima, Yoshinao Oda, Masafumi Nakamura

**Affiliations:** 1 Department of Surgery and Oncology, Graduate School of Medical Sciences, Kyushu University, 3-1-1 Maidashi, Higashi-ku, Fukuoka 812-8582, Japan; Department of Surgery and Oncology, Graduate School of Medical Sciences, Kyushu University, Fukuoka, Japan.; 2 Pancreatobiliary Surgery/ Kidney and Pancreas Transplantation, Kyushu University Hospital, 3-1-1 Maidashi, Higashi-ku, Fukuoka 812-8582, Japan.; 3 Department of Otorhinolaryngology Graduate School of Medical Sciences Kyushu University Fukuoka, Japan.; 4 Department of Respiratory Surgery, Hamanomachi Hospital, Fukuoka, Japan.; 5 Yakuin Internal Medicine and Cardiology Clinic, Fukuoka, Japan.; 6 Department of Respiratory Surgery, Japan Community Health Care Organisation, Kyushu Hospital, Kitakyushu, Japan.; 7 Fukuoka General Cancer Clinic, Fukuoka, Japan.; 8 Department of Pathology, Japanese Red Cross Fukuoka Hospital, Fukuoka, Japan.; 9 Department of Surgery and Oncology, Graduate School of Medical Sciences, Kyushu University, Fukuoka, Japan; Japanese Red Cross, Fukuoka Hospital, Fukuoka, Japan.; 10 Department of Anatomical Pathology, Graduate School of Medical Sciences, Kyushu University, Fukuoka, Japan.

## Abstract

Hypoxia is a critical feature of the tumour microenvironment in small cell lung cancer (SCLC) and contributes to malignant progression through hypoxia-inducible factor 1 alpha (HIF-1α)-mediated transcriptional programs. However, the upstream regulators that maintain HIF-1α stability under hypoxic conditions remain incompletely understood. In this study, we identified the chromosome 4 open reading frame 3 (C4orf3) as a hypoxia-inducible gene and investigated its functional significance in SCLC. C4orf3 expression is upregulated under hypoxic conditions, and its knockdown suppresses cell proliferation, migration, and invasion *in vitro* and reduces tumour growth *in vivo*. Mechanistically, C4orf3 depletion decreased HIF-1α protein levels even under chemically induced hypoxia, suggesting that its regulation is independent of the canonical PHD-VHL degradation pathway. Further analysis demonstrated that C4orf3 modulates HIF-1α stability through PIASy-mediated SUMOylation. Clinical relevance was supported by a positive association between C4orf3 and HIF-1α expression in resected SCLC tissues. These findings suggested that C4orf3 functions as a regulator of hypoxic adaptation in SCLC by maintaining HIF-1α stability and may represent a potential therapeutic target in hypoxia-driven tumour progression.

## Introduction

Small cell lung cancer (SCLC) is an aggressive neuroendocrine malignancy that accounts for approximately 10-15% of all lung cancers. Despite initial sensitivity to chemotherapy and radiotherapy, most patients relapse rapidly, and overall survival remains poor. The incidence rate in the United States was approximately 4.7 per 100,000 individuals in 2021, and the 3-year survival rates remain 56.5% for limited-stage disease and 17.6% for extensive-stage disease 1. These clinical features highlight the urgent need to identify novel molecular determinants of SCLC progression. Several molecular classifiers, including ASCL1, NEUROD1, POU2F3, and YAP1, have been proposed as prognostic biomarkers for SCLC 2.

Tumour hypoxia is a key driver of malignant progression in solid tumours and promotes proliferation, survival, invasion, and metastasis. In SCLC, hypoxia-inducible factor 1 alpha (HIF-1α) is a central mediator of the hypoxic response and has been reported to correlate with poor prognosis in patients with high HIF-1α expression 3,4. These findings indicate that hypoxic signalling contributes substantially to SCLC pathobiology; however, the upstream molecular regulators that sustain HIF-1α stability under hypoxic conditions remain poorly defined.

We have previously shown that several hypoxia-inducible molecules, including liprin-α4, MAML3, RBPL, FAM115C, and C4orf47, are upregulated in pancreatic cancer under hypoxic conditions 5-8, although none have yet been translated into clinical application. Recently, our group identified the chromosome 4 open reading frame 3 (C4orf3) as a hypoxia-inducible gene through transcriptomic screening. C4orf3 is an endoplasmic reticulum-associated transmembrane protein that regulates Ca²⁺-ATPase activity in adipose tissue 9; however, its function in cancer biology has not been elucidated in any malignancy, including SCLC.

In this study, we aimed to clarify the biological role of C4orf3 in SCLC, determine whether its hypoxia-induced expression contributes to tumour progression, and explore its potential as a novel therapeutic target.

## Materials and Methods

### Cell culture and reagents

Three human SCLC cell lines (SBC-5, S2, and 87-5) were used in this study. Cells were cultured in RPMI-1640 medium supplemented with 10% foetal calf serum (FCS; Life Technologies, Grand Island, NY, USA), 100 U/mL penicillin, and 100 μg/mL streptomycin (Nacalai Tesque, Kyoto, Japan). The cells were maintained under normoxic conditions (5% CO₂ and 95% ambient air). To establish hypoxic culture conditions, the cells were incubated in a multi-gas incubator (Sanyo, Tokyo, Japan) at 1% O₂, 5% CO₂, and 94% N₂. To induce chemical hypoxia, 100 μM cobalt (II) chloride (CoCl₂; FUJIFILM Wako Pure Chemical, Osaka, Japan) was added to the culture medium under normoxic conditions.

### DNA microarray

Total RNA was amplified, labelled, and hybridised according to the manufacturer's instructions (Agilent, Santa Clara, CA, USA). Relative hybridisation intensities and background values were calculated using the Agilent Feature Extraction software. Normalised signal intensities were converted into Z-scores and ratios, and the gene expression profiles of ASPC-1 cells cultured under hypoxic conditions for 48 h were compared to those of cells maintained under normoxia. The same analysis was performed using the SUIT-2 cells. In addition, SBC-5 cells transfected with either control or C4orf3 siRNA were cultured under hypoxic conditions and analysed using the same protocol and statistical pipeline. Gene set enrichment analysis (GSEA) was performed using the Metascape platform 10.

### Western blotting and immunoprecipitation analyses

Western blotting was performed as described previously 11. After electrophoretic transfer, the membranes were incubated with primary antibodies against C4orf3 (no. PA5-113166; Thermo Fisher Scientific, Waltham, MA, USA), HIF-1α (no. D1S7W; Cell Signaling Technology, Danvers, MA, USA), PIASy (no. C-11; Santa Cruz Biotechnology, Dallas, TX, USA), TWIST (no. sc-81414; Santa Cruz Biotechnology), SNAI1 (No. sc-271977; Santa Cruz Biotechnology), SLUG (no. sc-166476; Santa Cruz Biotechnology), and SUMO1 (no. sc-5308; Santa Cruz Biotechnology) for 1 h at room temperature (20-25 °C) followed by overnight incubation at 4 °C. After washing, the membranes were incubated for 3 h at room temperature (20-25 °C) with horseradish peroxidase-conjugated secondary antibodies (anti-mouse IgG, No. NA931; and anti-rabbit IgG, no. NA934; Amersham Biosciences, Cytiva). Immunoreactive bands were visualised using Amersham ECL Prime Western Blotting Detection Reagent (GE Healthcare, Tokyo, Japan) and captured using an EZ Capture ST imaging system (ATTO, Tokyo, Japan). α-Tubulin (Sigma-Aldrich, St. Louis, MO, USA) was used as the loading control. Immunoprecipitation was performed using the Dynabeads Protein G Immunoprecipitation Kit (no. DB10007; Invitrogen, Waltham, MA, USA) according to the manufacturer's instructions. Briefly, antibodies were incubated with Dynabeads Protein G to generate antibody-bead complexes. The complexes were then incubated with cell lysates at 4 °C for 1 h with gentle rotation. For immunoprecipitation, an anti-HIF-1α antibody (no. D1S7W; Cell Signaling Technology) was used. After washing to remove non-specifically bound proteins, the immune complexes were eluted for subsequent analysis.

### Real-time PCR

Total RNA was extracted from SBC-5, S2, and 87-5 cells using the High Pure RNA Isolation Kit (Roche, Mannheim, Germany) and quantified with a spectrophotometre (Ultraspec 2100; Amersham Pharmacia Biotech, Cambridge, UK). One microgram of RNA was treated with DNase and reverse-transcribed into cDNA using the QuantiTect Reverse Transcription Kit (Qiagen, Valencia, CA, USA) following the manufacturer's protocol. Quantitative real-time PCR was performed on a StepOnePlus Real-Time PCR System (Applied Biosystems, Carlsbad, CA, USA) using iQ SYBR Green Supermix (Bio-Rad).

All reactions were performed in triplicate, and each experiment was independently repeated three times. A cycle threshold (Ct) cutoff of 37 was used to define detectable expression; therefore, only samples with Ct < 37 were included in the analysis. Primer sequences for human C4orf3 were purchased from Thermo Fisher Scientific (No. A15629 and No. A15630). β-Actin was used as the internal control, with the primer sequences 5′-TTGCCGACAGGATGCAGAAGGA-3′ and 5′-AGGTGGACAGCGAGGCCAGGAT-3′. Relative gene expression was calculated after normalisation to β-actin.

### Immunocytochemistry

SBC-5 cells were seeded onto glass coverslips and placed in 12-well culture plates, then allowed to adhere for 24 h. After two washes with phosphate-buffered saline (PBS), the cells were fixed in 4% paraformaldehyde in PBS for 10 min, then rinsed three times with PBS. Permeabilisation was performed using 0.5% Triton X-100 in PBS for 5 min, followed by three additional washes with PBS. The samples were then blocked with Blocking One-P (Nacalai Tesque) for 15 min and incubated overnight at 4°C with primary antibodies against C4orf3 (207622-T08; Sino Biological, Tokyo, Japan) and HIF-1α (No. D5F3M; Cell Signalling Technology) diluted in 10% Blocking One-P. After washing, Alexa Fluor 488-conjugated donkey anti-rabbit IgG (1:50; Invitrogen) and Alexa Fluor 555-conjugated donkey anti-mouse IgG (1:50; Invitrogen) diluted in 10% Blocking One-P were applied for 40 min at room temperature (20-25 °C). The coverslips were then washed and mounted on glass slides using the ProLong Diamond Antifade Reagent with DAPI (Thermo Fisher Scientific). Images were acquired using a Nikon A1 confocal laser-scanning microscope.

### Cell invasion assay

Cell invasiveness was evaluated using a Matrigel-coated Transwell invasion system as described previously (Matsushita et al., 2014). Briefly, siRNA-transfected cells (2.0 × 105) were seeded in the upper compartment and maintained under normoxic conditions for 18 h. The cells that migrated to the lower chamber through the Matrigel matrix were fixed and counterstained with DAPI (Thermo Fisher Scientific). The number of invading cells was quantified at ×200 magnification using a Nikon Eclipse TE300 microscope.

### Cell proliferation assay

Cell proliferation was assessed using Cell Count Reagent SF (Nacalai Tesque). SBC-5, S2, and 87-5 cells were transfected with either C4orf3 siRNA or negative control siRNA and seeded into 96-well plates at a density of 4.0 × 10³ cells/well. The cells were incubated at 37 °C for 0, 24, 48, or 72 h, after which Cell Count Reagent SF was added and the plates were incubated for an additional 1 h at 37 °C. The absorbance was measured at 492 nm with a reference wavelength of 620 nm using a Biotrak Visible Plate Reader (Amersham Biosciences; Cytiva). Cell viability was calculated relative to the 0 h time point.

### Apoptosis assay

Apoptosis was assessed using the Annexin V-FITC Apoptosis Detection Kit (Nacalai Tesque), according to the manufacturer's instructions. Briefly, siRNA-transfected cells were harvested, washed twice with ice-cold PBS, and resuspended in 1× binding buffer. The cell suspension was incubated with Annexin V-FITC and propidium iodide for 15 min at room temperature (20-25 °C) in the dark. Fluorescence signals were acquired using a FACScan flow cytometer (BD Biosciences, San Jose, CA, USA), and data were analysed with BD CellQuest Pro software version 6.0 (BD Biosciences). A total of 20,000 events per sample were collected, and apoptotic cells were quantified as the proportion of Annexin V-positive cells.

### *In vivo* xenograft tumour model

Four-week-old female BALB/c nu/nu athymic nude mice were obtained from Charles River Laboratories Japan (Kanagawa, Japan) and acclimated for 1 week before experimentation. All animal experiments were reviewed and approved by the Animal Welfare Committee of Kyushu University (approval no. A24-094-0), and performed in accordance with the “Guidelines for the Proper Conduct of Animal Experiments” issued by the Science Council of Japan. Mice were housed in a specific pathogen-free facility under controlled environmental conditions (temperature 26-28 °C, relative humidity 40-70%, and a 12-h light/dark cycle from 8:00 a.m. to 8:00 p.m.), with free access to food and water. The number of animals used was minimised, and humane care was provided throughout the study. Euthanasia criteria were defined as a tumour diameter exceeding 10 mm, persistent distress, or skin ulcerations at the graft site. Mice meeting these criteria were sacrificed using an inhalational overdose of sevoflurane, and death was verified by the absence of cardiopulmonary activity and pupil reflex. SBC-5 cells transfected with either C4orf3 siRNA or control siRNA (1.0 × 10^6^ cells in Matrigel per animal) were injected subcutaneously into both inguinal regions (n = 4 mice/group). Tumour dimensions were recorded every 2 days, and tumour volume was calculated using the formula L × D^2^, where L represents the longest tumour diameter, and D is the shortest perpendicular diameter.

### Immunohistochemistry

Paraffin-embedded tumour specimens were collected from five patients with SCLC who underwent surgical resection at the Japanese Red Cross Fukuoka Hospital (Fukuoka, Japan) between 2021 and 2023. Written informed consent for the use of clinical samples was obtained from all participants. Sections of 4 μm thickness were de-paraffinised, rehydrated, and subjected to immunohistochemical staining using primary antibodies against C4orf3 (No. 207622-T08; Sino Biological) and HIF-1α (No. ab51608; Abcam, Cambridge, UK). Endogenous peroxidase activity was quenched using 3% hydrogen peroxide for 5 min. Heat-induced epitope retrieval was performed in the Target Retrieval Solution (pH 9.0; Agilent Technologies) for 10 min under high pressure. Samples were incubated with primary antibodies overnight at 4 °C and then treated with secondary antibodies for 40 min at room temperature (20-25 °C). Immunoreactivity was visualised using the HISTOFINE Simple Stain MAX-PO (R) system (Nichirei, Tokyo, Japan) with diaminobenzidine as the chromogen, followed by haematoxylin counterstaining. Positive and negative controls were included. The number of positively stained tumour-infiltrating cells was manually counted.

### Statistical analyses

Quantitative data are expressed as the mean ± standard deviation (SD). Differences between the two groups were evaluated using an unpaired Student's t-test. All the analyses were performed using Microsoft Excel (Microsoft). Statistical significance was set at p < 0.05.

## Results

### C4orf3 expression is induced under hypoxic conditions in SCLC cells

**Figure 1:**
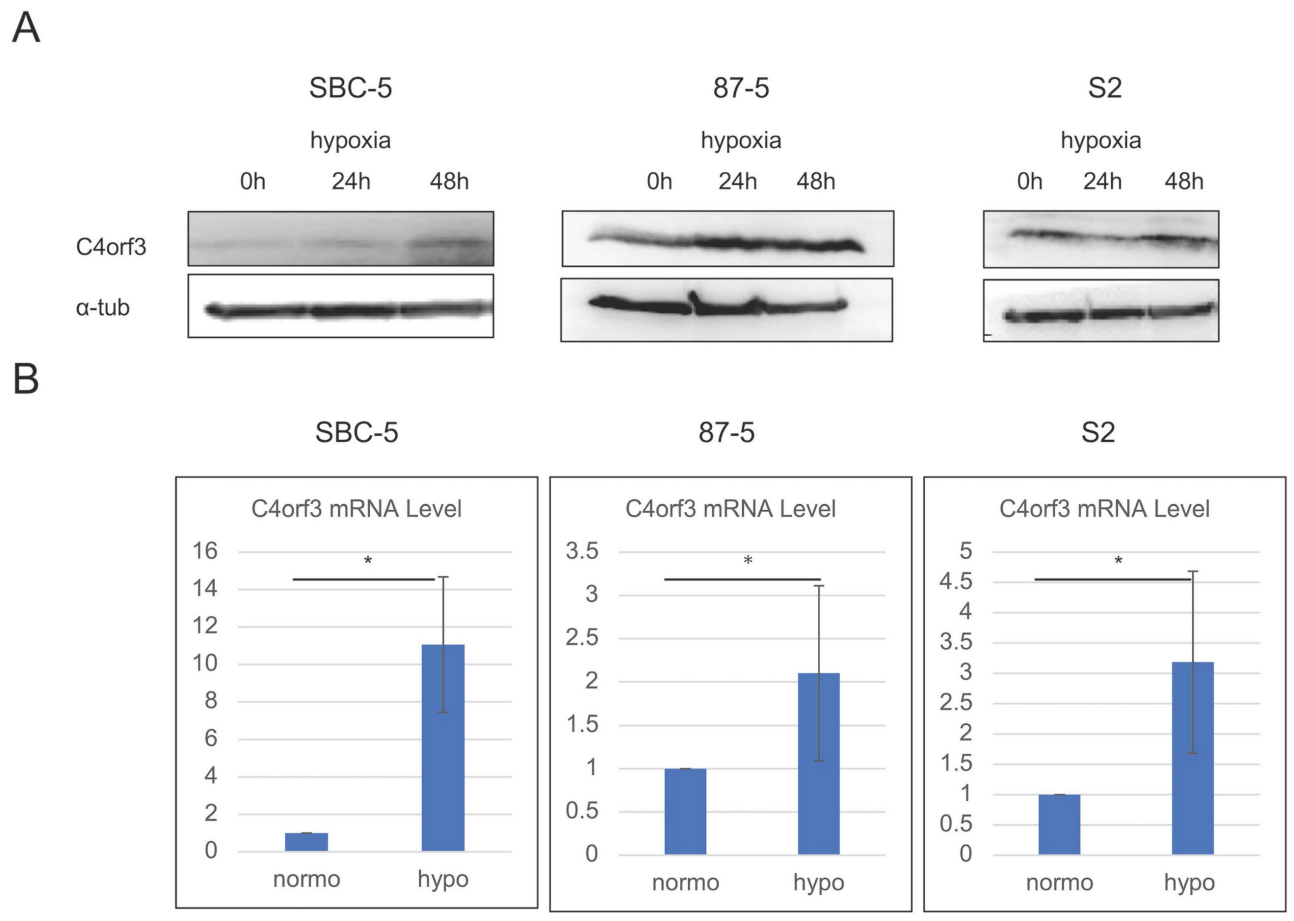
C4orf3 expression is upregulated under hypoxic conditions in SCLC cell lines. (A) Western blotting analysis showing increased C4orf3 protein levels after 0, 24, and 48 h of hypoxic culture. (B) Real-time PCR analysis demonstrating increased C4orf3 mRNA expression after 48 h of hypoxia compared with normoxia. *p < 0.05; error bars represent SD.

To identify hypoxia-inducible genes, we first performed a microarray analysis using pancreatic cancer cell lines (ASPC-1 and SUIT-2) cultured under hypoxic and normoxic conditions. Through this screening, C4orf3 was identified as one of the genes markedly upregulated under hypoxia compared to normoxia (Table 1), and this induction was confirmed at the protein level in SUIT-2 cells Fig. S1. Next, we examined whether a similar regulation occurs in SCLC. SBC-5, S2, and 87-5 cells were cultured under hypoxic conditions for up to 48 h, and C4orf3 expression was assessed using western blotting and real-time PCR. In all three SCLC cell lines, hypoxia significantly increased C4orf3 protein expression compared to normoxic conditions (Fig. 1A), and a corresponding increase in C4orf3 mRNA levels was also observed (Fig. 1B). These findings indicated that hypoxia induced C4orf3 expression in SCLC cells.

### C4orf3 knockdown suppresses SCLC cell proliferation under normoxic and hypoxic conditions

**Figure 2:**
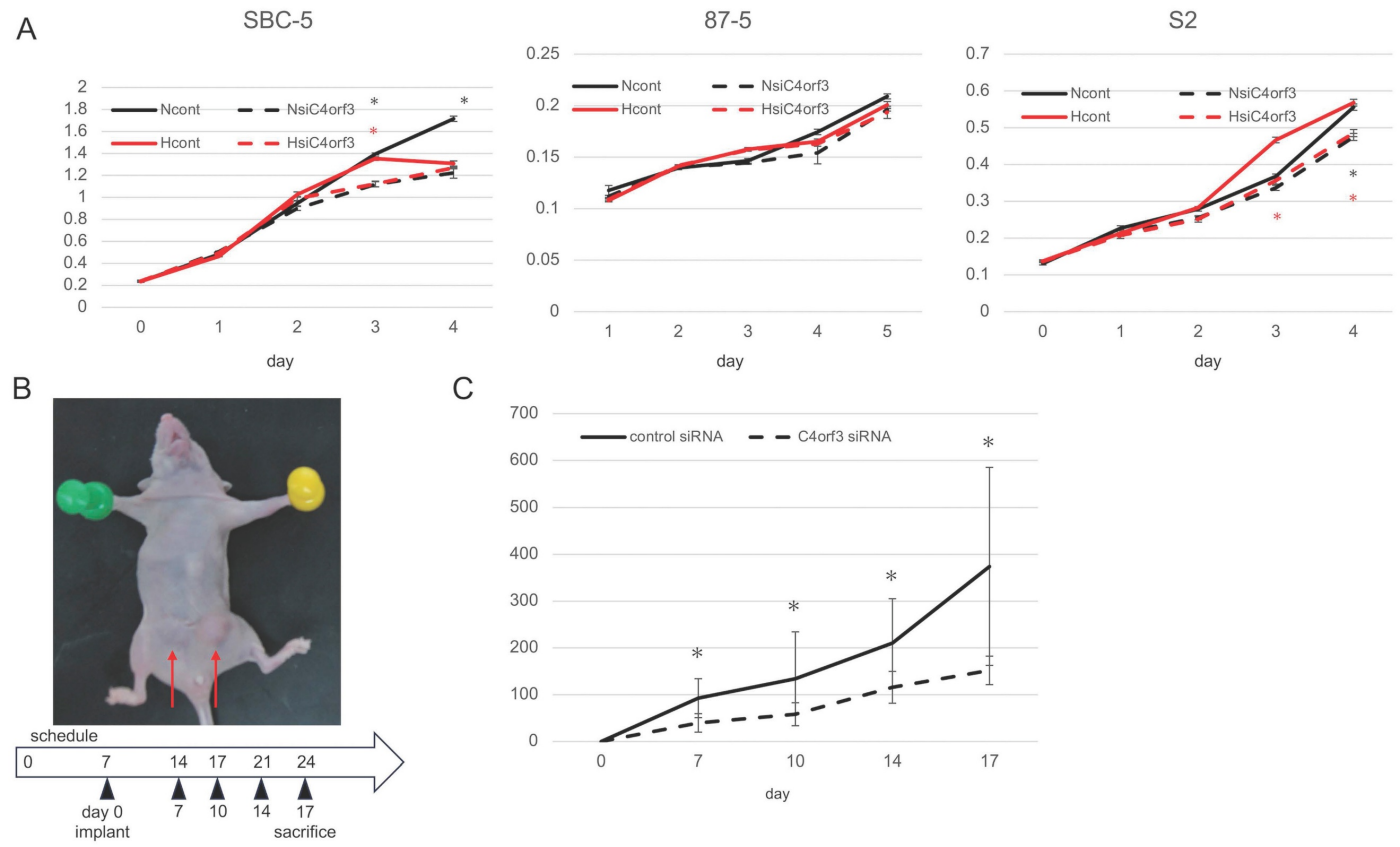
C4orf3 promotes tumour growth *in vitro* and *in vivo*. (A) Proliferation of SCLC cell lines transfected with control or C4orf3 siRNA under normoxic (N) or hypoxic (H) conditions. (B) Subcutaneous xenograft model generated by implanting SBC-5 cells transfected with control or C4orf3 siRNA into the inguinal region of female nude mice (BALB/c nu/nu) (n = 4 per group). (C) Tumour volume measurements over time, collected at the end of the experiment. *p < 0.05; error bars represent SD.

To investigate whether C4orf3 contributes to tumour cell growth, we examined the effect of C4orf3 knockdown on the proliferation of SCLC cell lines. Three independent siRNAs targeting C4orf3 were transfected into SBC-5, S2, and 87-5 cells, and cell proliferation was assessed under normoxic and hypoxic conditions. In SBC-5 and S2 cells, C4orf3 silencing reduced proliferation under normoxia, whereas 87-5 cells did not exhibit a significant reduction in proliferation under either condition (Fig. 2A).

To evaluate the relevance of these findings *in vivo*, SBC-5 cells transfected with either control or C4orf3 siRNA were implanted into both the inguinal regions of thymus-free nude mice (Fig. 2B). Tumour growth was significantly suppressed in the C4orf3 knockdown group compared to that in the control group (Fig. 2C).

### C4orf3 knockdown reduces HIF-1α expression under hypoxic conditions

**Figure 3:**
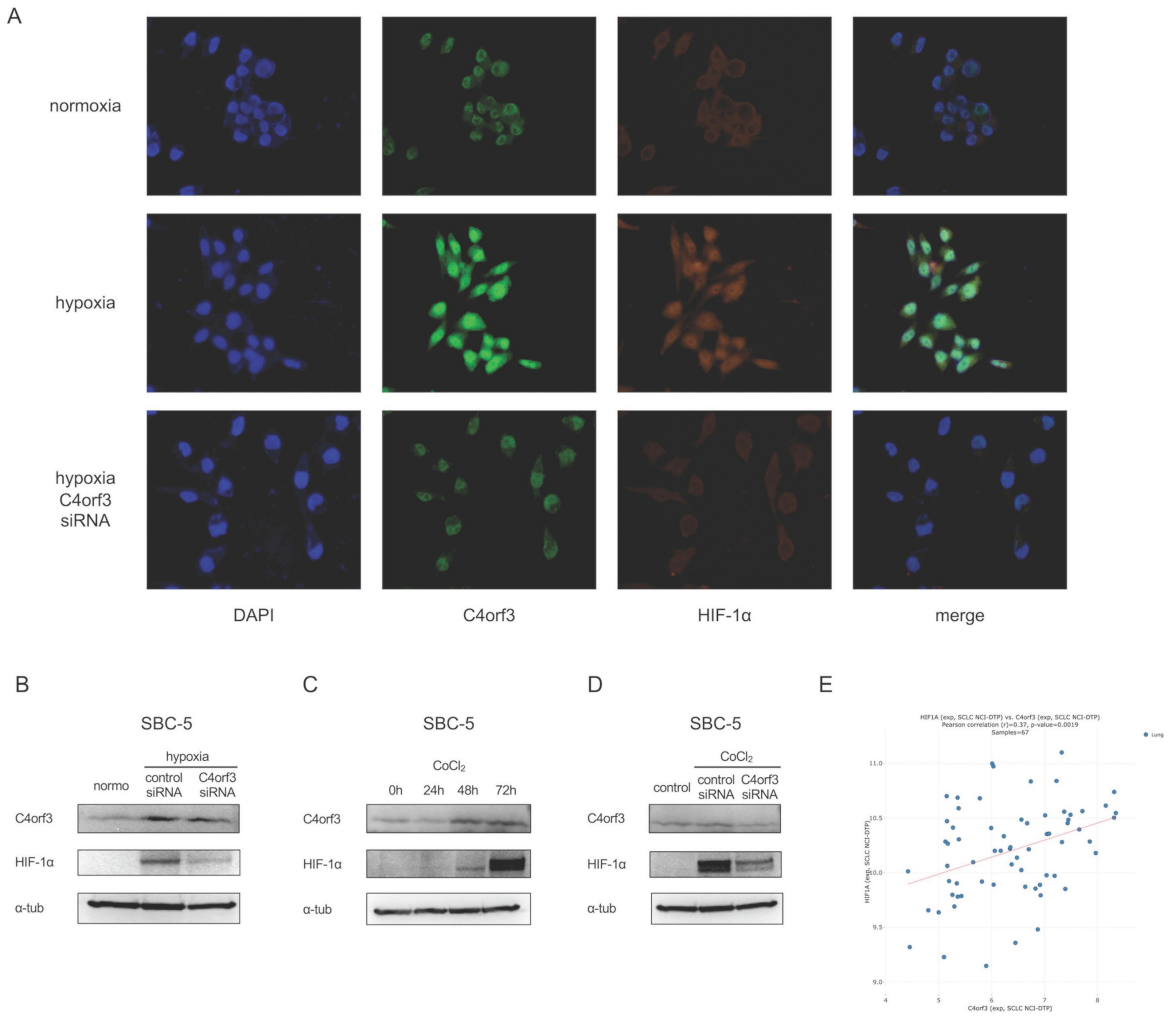
C4orf3 knockdown decreases HIF-1α expression in SCLC cells. (A) Immunofluorescence staining of SBC-5 cells showing perinuclear localisation of C4orf3 (green) and decreased HIF-1α (red) expression under hypoxia following C4orf3 knockdown. Nuclei were counterstained with DAPI (blue). (B) Western blotting analysis of C4orf3 and HIF-1α in SBC-5 cells cultured under hypoxic conditions with control or C4orf3 siRNA. (C) Western blotting analysis showing CoCl₂-induced upregulation of C4orf3 and HIF-1α under normoxia. (D) Western blotting analysis demonstrating attenuation of CoCl₂-induced HIF-1α expression following C4orf3 knockdown. (E) Positive correlation between C4orf3 and HIF-1α mRNA expression in SCLC cell lines based on the CellMiner-SCLC dataset.

To clarify the intracellular localisation of C4orf3, we performed immunocytochemistry on SBC-5 cells. C4orf3 was predominantly detected in the perinuclear region, and its expression increased under hypoxic conditions (Fig. 3A). Interestingly, when C4orf3 was silenced under hypoxia, HIF-1α expression was simultaneously reduced.

We examined this finding in more detail using western blotting analysis. Consistent with the immunocytochemistry results, C4orf3 knockdown led to a marked reduction in HIF-1α protein levels in SBC-5 cells cultured under hypoxic conditions (Fig. 3B).

We used a chemically induced hypoxia model to validate this regulatory relationship. Treatment of SBC-5 cells with cobalt chloride (CoCl₂) increased HIF-1α expression in a time-dependent manner, accompanied by a parallel increase in C4orf3 (Fig. 3C). In contrast, when C4orf3 was silenced in CoCl₂-treated cells, the induction of HIF-1α was markedly attenuated (Fig. 3D). Analysis using the CellMiner-SCLC database 12 also demonstrated a positive correlation between C4orf3 and HIF-1α expression (Fig. 3E). These findings indicated that C4orf3 positively regulated HIF-1α expression under hypoxic conditions.

### C4orf3 regulates HIF-1α through PIASy-mediated SUMOylation

HIF-1α is normally degraded under normoxia via hydroxylation by prolyl hydroxylase domain proteins (PHDs), followed by ubiquitination by von Hippel-Lindau (VHL) and proteasomal degradation. Because the reduction in HIF-1α observed after C4orf3 knockdown persisted even in the presence of CoCl₂, which inhibits PHD activity 13, we focused on the alternative PIASy-mediated regulatory pathway. PIASy promotes SUMOylation of HIF-1α, thereby negatively regulating its stability 14.

**Figure 4:**
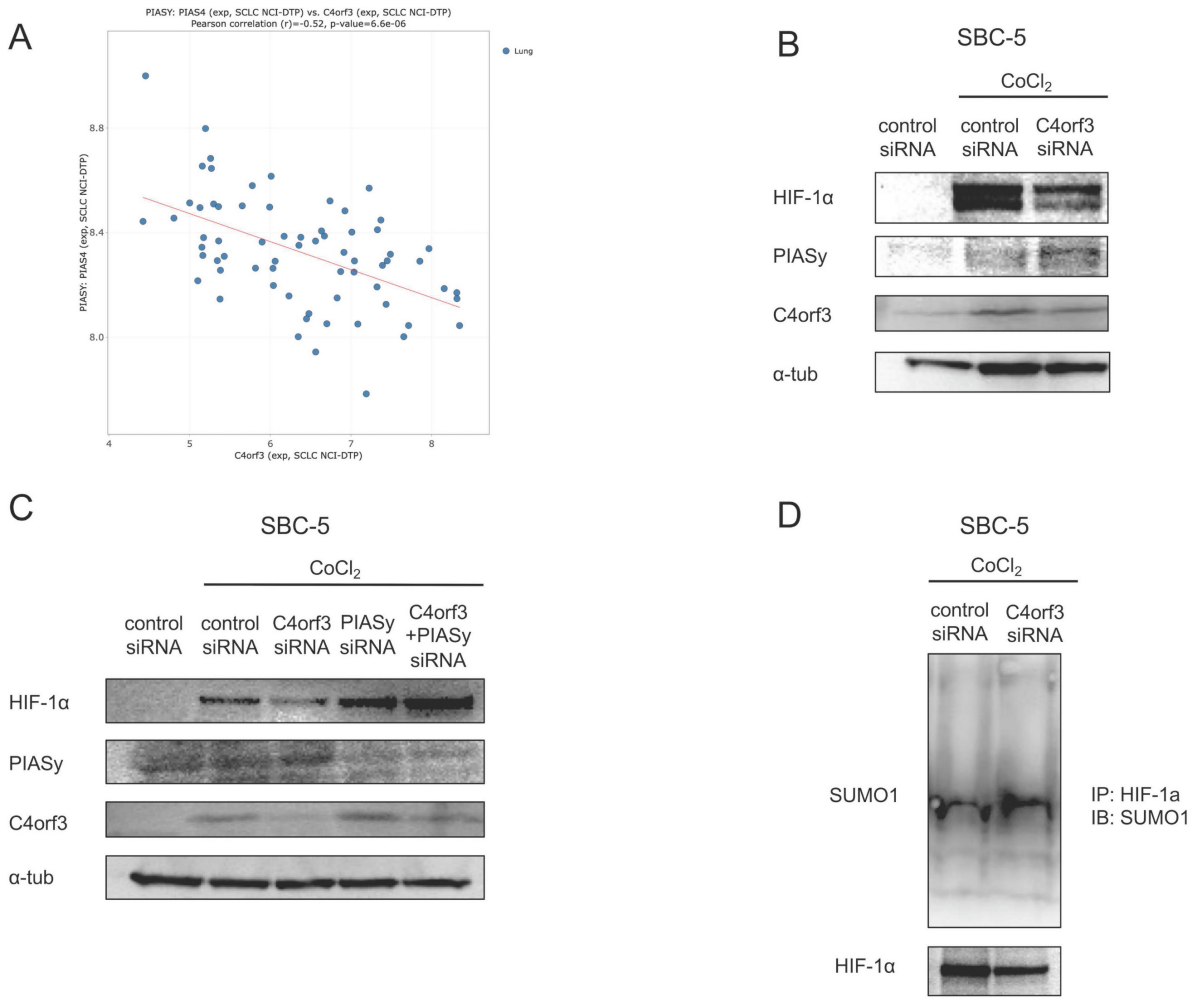
C4orf3 regulates HIF-1α stability through PIASy-mediated SUMOylation. (A) Negative correlation between C4orf3 and PIASy mRNA expression based on the CellMiner-SCLC dataset. (B) Western blotting analysis showing increased PIASy expression following C4orf3 knockdown in CoCl₂-treated SBC-5 cells. (C) Rescue of HIF-1α expression by simultaneous knockdown of PIASy in C4orf3-silenced SBC-5 cells under CoCl₂ treatment. (D) Immunoprecipitation analysis demonstrating increased SUMO1 binding to HIF-1α following C4orf3 knockdown.

Using the CellMiner-SCLC database, we observed a negative correlation between C4orf3 and PIASy expression (Fig. 4A). Consistent with this, the silencing of C4orf3 in SBC-5 cells resulted in increased PIASy protein levels (Fig. 4B). Furthermore, simultaneous knockdown of C4orf3 and PIASy restored HIF-1α expression that had been reduced by C4orf3 inhibition alone (Fig. 4C). To confirm whether this mechanism involves enhanced SUMOylation of HIF-1α, we performed immunoprecipitation analysis and observed increased binding between HIF-1α and SUMO1 following C4orf3 knockdown (Fig. 4D). Taken together, these findings suggested that C4orf3 regulated HIF-1α stability by modulating PIASy-mediated SUMOylation under hypoxic conditions.

### C4orf3 expression correlates with HIF-1α in human SCLC specimens

**Figure 5:**
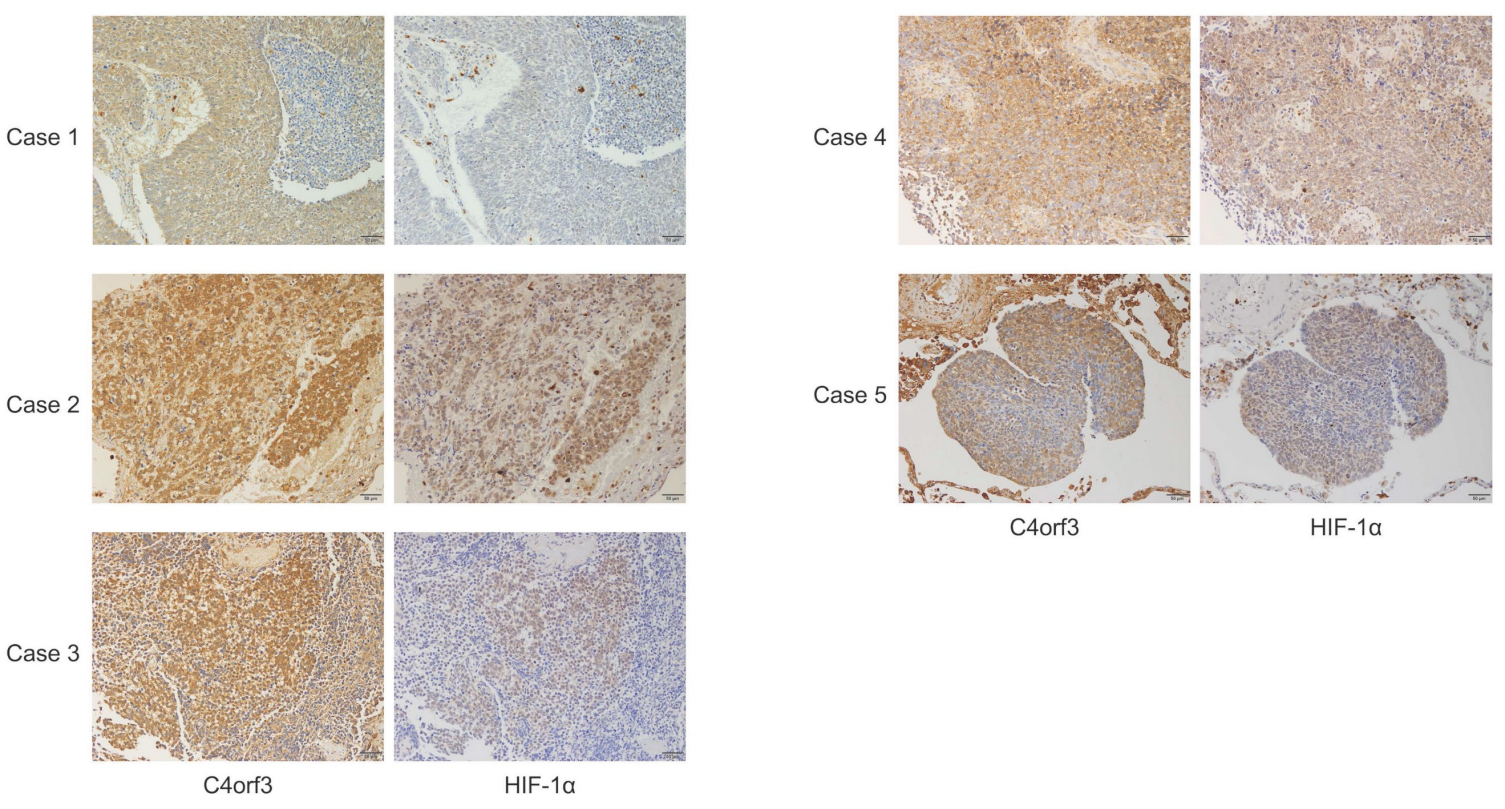
Immunohistochemical analysis of C4orf3 and HIF-1α expression in human SCLC specimens. Representative staining patterns from five clinical cases (×200 magnification).

To evaluate the relevance of these findings to human disease, immunohistochemical analysis was performed using surgically resected specimens from four patients with SCLC and one mediastinal lymph node biopsy specimen (Fig. 5). The clinicopathological characteristics of the five patients are summarised in Table 2. C4orf3 expression was observed in both normal lung tissues (alveolar and bronchiolar epithelium) and SCLC tumour tissues, with a predominant cytoplasmic localisation. The intensity of C4orf3 staining varied among the cases, and no clear association with the surgical procedure or disease stage was observed.

In contrast, tumours with stronger C4orf3 expression tended to show higher HIF-1α expression. In cases 2 and 3, focal regions with high C4orf3 expression exhibited concordant HIF-1α upregulation. By contrast, case 1 showed detectable C4orf3 but little to no HIF-1α staining. Because this case underwent a more prolonged surgical procedure (lobectomy) than the others, delayed fixation may have led to post-excisional degradation of HIF-1α, which is rapidly turned over. In cases 4 and 5, overall C4orf3 expression was relatively weak; however, localised regions with stronger C4orf3 staining also showed concurrent HIF-1α positivity.

These findings suggested that C4orf3 expression in human SCLC tissue was positively associated with HIF-1α expression, particularly in regions likely to reflect a hypoxic microenvironment.

### C4orf3 promotes malignant phenotypes of SCLC through HIF-1α

**Figure 6:**
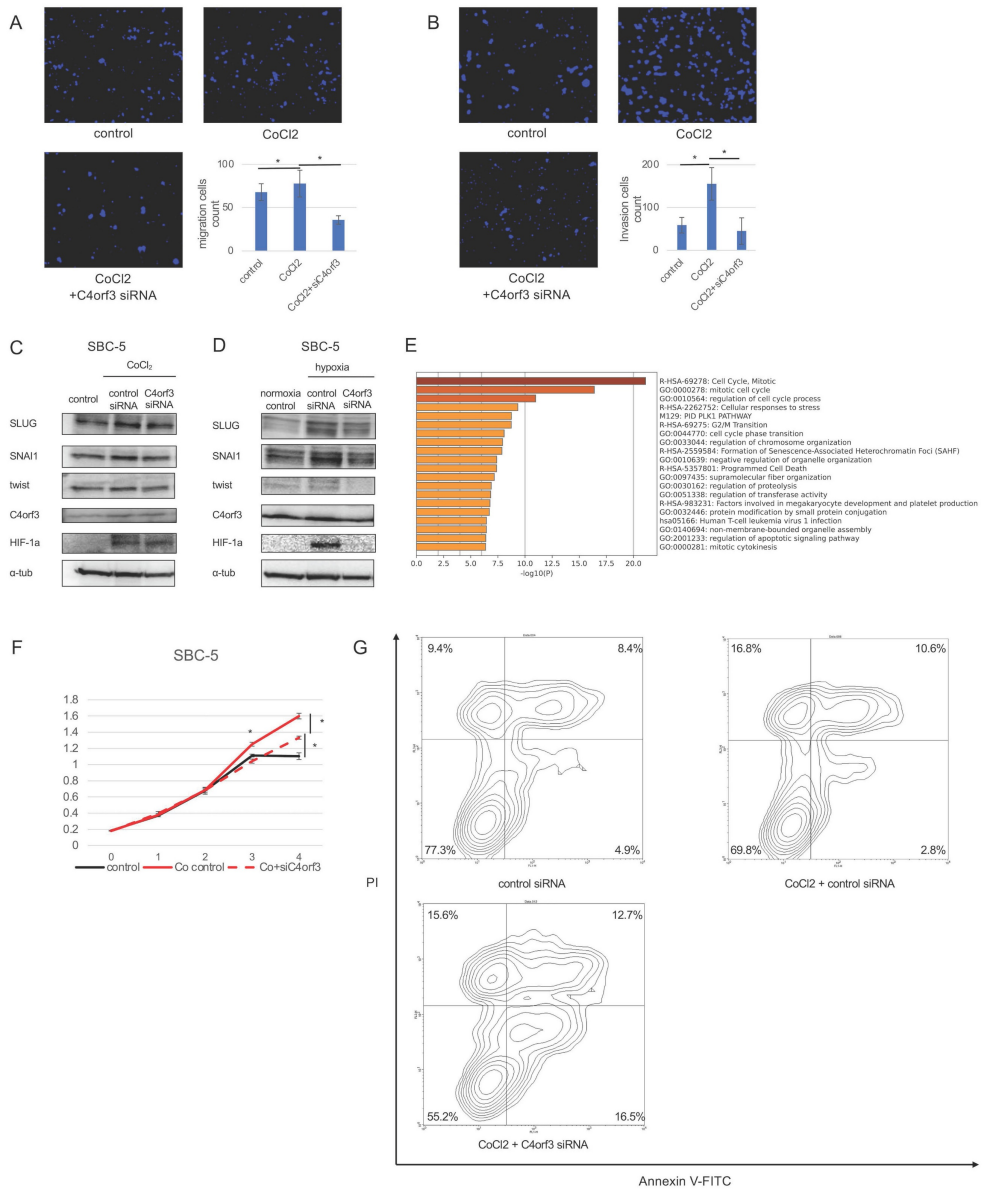
C4orf3 contributes to malignant phenotypes of SCLC through HIF-1α. (A,B) Migration and invasion assays of SBC-5 cells treated with CoCl₂ following transfection with control or C4orf3 siRNA. Nuclei were counterstained with DAPI (blue). *p < 0.05; error bars represent SD. (C,D) Western blotting analysis showing hypoxia- or CoCl₂-induced upregulation of EMT-related markers (SLUG, SNAI1, and TWIST) and attenuation of their expression following C4orf3 knockdown. (E) Gene set enrichment analysis of SBC-5 cells cultured under hypoxic conditions for 48 h, with or without C4orf3 knockdown. (F) Proliferation assay of SBC-5 cells cultured in CoCl₂ showing reduced growth following C4orf3 knockdown. (G) Apoptosis assay showing an increased apoptotic fraction after C4orf3 silencing following CoCl₂ treatment.

To determine whether C4orf3-mediated regulation of HIF-1α contributes to the malignant phenotype of SCLC cells, we evaluated cellular migration and invasion under conditions of enforced HIF-1α expression. SBC-5 cells were treated with CoCl₂ to stabilise HIF-1α and subsequently transfected with siC4orf3. Forced expression of HIF-1α enhanced the migratory capacity of SBC-5 cells, whereas silencing of C4orf3 attenuated this effect (Fig. 6A, 6B).

Next, we assessed epithelial-mesenchymal transition (EMT)-related markers. Western blotting analysis demonstrated that CoCl₂ treatment upregulated SLUG, TWIST, and SNAI1, consistent with HIF-1α activation, whereas C4orf3 knockdown reduced the expression of all three markers (Fig. 6C). Similar results were obtained when SBC-5 cells were cultured under hypoxic conditions, where C4orf3 inhibition suppressed the hypoxia-induced upregulation of SLUG, TWIST, and SNAI1 (Fig. 6D).

To further characterise the downstream functional consequences, we performed microarray analysis of SBC-5 cells exposed to hypoxia with or without C4orf3 knockdown. GSEA revealed enrichment of pathways related to cell cycle regulation and programmed cell death among the differentially expressed genes. Functionally, CoCl₂ treatment enhanced cell proliferation compared to controls, whereas C4orf3 knockdown restored proliferative activity to baseline levels (Fig. 6E). Flow cytometry confirmed that silencing C4orf3 increased the proportion of apoptotic cells (Fig. 6F).

Taken together, these findings indicated that C4orf3 contributed to the malignant phenotype of SCLC cells by sustaining HIF-1α-driven migration, EMT activation, and resistance to apoptosis.

## Discussion

In this study, we demonstrated that C4orf3 is upregulated under hypoxic conditions in SCLC cells and that its knockdown suppresses tumour growth both *in vitro* and *in vivo*. Importantly, silencing C4orf3 reduced HIF-1α expression even under chemically induced hypoxia, suggesting that this regulation is independent of the canonical PHD-VHL degradation pathway. Instead, our findings indicated that C4orf3 modulated HIF-1α stability through a PIASy-mediated mechanism.

HIF-1α is normally degraded under normoxia through hydroxylation by PHDs followed by VHL-dependent ubiquitination. However, the persistence of HIF-1α suppression despite CoCl₂ treatment indicated the involvement of a non-PHD degradation mechanism. Therefore, we focused on PIASy, which promotes SUMOylation of HIF-1α and negatively regulates its stability. Consistent with this, C4orf3 knockdown increased PIASy expression and enhanced SUMO1 binding to HIF-1α, while simultaneous knockdown of PIASy restored HIF-1α protein levels. These results suggested that C4orf3 maintained HIF-1α stability by suppressing PIASy-mediated SUMOylation under hypoxic conditions.

**Figure 7:**
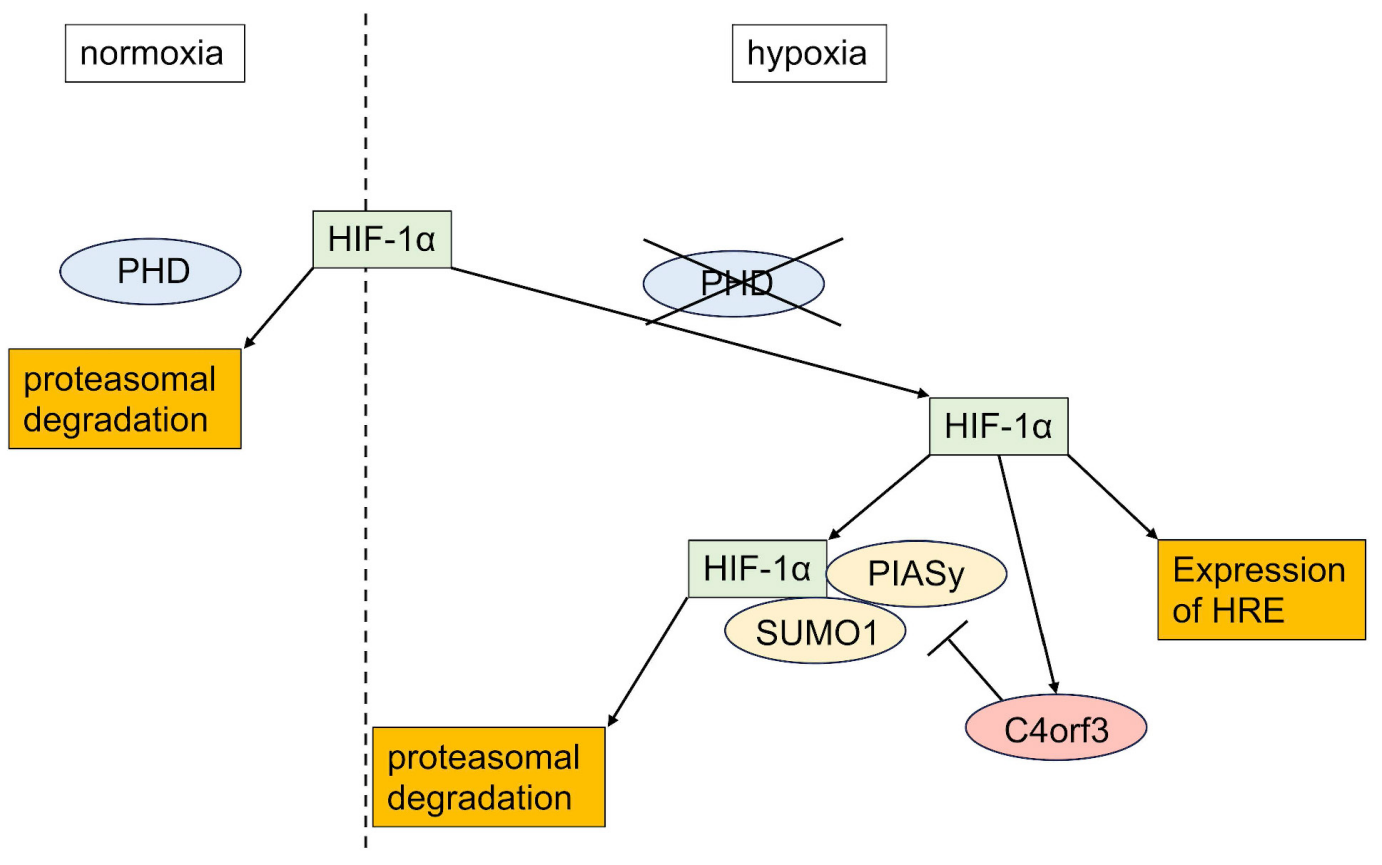
Proposed model of C4orf3-mediated regulation of HIF-1α in SCLC. Under hypoxic conditions, C4orf3 suppresses PIASy-mediated SUMOylation, thereby stabilising HIF-1α and promoting malignant phenotypes such as proliferation, migration, and invasion in SCLC cells.

Although the present study focused on SUMOylation-mediated regulation, C4orf3 may exert broader biological effects beyond hypoxia-dependent stabilisation of HIF-1α. Notably, C4orf3 knockdown suppressed tumour growth even under normoxic conditions, suggesting that C4orf3 may influence additional malignant pathways independent of hypoxic signalling. This observation is consistent with reports on other malignancies, including clear cell renal cell carcinoma and colorectal cancer, in which C4orf3 has been implicated 15,16. Because ccRCC is characterised by constitutive HIF-1α activation due to loss-of-function mutations in pVHL 17, the present findings further supported a link between C4orf3 and HIF-1α regulation in solid tumours. A schematic overview of this mechanism is illustrated in Figure 7, summarising the role of C4orf3 as a hypoxic regulator that stabilises HIF-1α by suppressing PIASy-mediated SUMOylation.

C4orf3 regulates Ca²⁺-ATPase activity by interacting with SERCA2b in adipose tissues 9. In parallel, Neumann et al. demonstrated that constitutive HIF-1α activation in thymocytes drives SERCA2 overexpression and alters Ca²⁺ handling, indicating potential reciprocal regulation between Ca²⁺ signalling and hypoxic adaptation 18. In addition, numerous studies have reported crosstalk between Ca²⁺-dependent pathways and HIF-1α signalling in several cancers 19-21. These findings raise the possibility that C4orf3 may function as a Ca²⁺-responsive regulator of HIF-1α stability in hypoxic tumour cells. Although our present work did not directly assess Ca²⁺ dynamics, the PIASy-mediated mechanism identified here does not exclude the involvement of Ca²⁺ signalling upstream or in parallel. Investigation of Ca²⁺-dependent regulation of C4orf3 remains an important subject for future studies.

Our clinical observations also supported a functional relationship between C4orf3 and HIF-1α *in vivo*. In surgically resected SCLC specimens, C4orf3 colocalised with HIF-1α in tumour regions, particularly where hypoxia is likely to be present. The absence of detectable HIF-1α in one case was plausibly attributable to delayed fixation, consistent with its rapid *ex vivo* degradation. Although the number of available resected SCLC specimens was limited due to the rarity of surgical candidates, the observed concordance between C4orf3 and HIF-1α expression is consistent with our mechanistic findings.

Collectively, these results suggested that C4orf3 contributed to hypoxia-driven malignant progression in SCLC by sustaining HIF-1α stability and may represent a previously unrecognised component of hypoxic adaptation in solid tumours.

In summary, this study demonstrated that C4orf3 was upregulated under hypoxic conditions in SCLC and that its depletion exerted tumour-suppressive effects. C4orf3 regulated HIF-1α stability through PIASy-mediated SUMOylation, thereby contributing to hypoxia-driven malignant phenotypes. These findings suggested that targeting C4orf3 may represent a novel therapeutic approach for SCLC and provide new insights into the molecular mechanisms linking hypoxic adaptation to tumour progression.

## Supplementary Material

Supplementary figure.

## Figures and Tables

**Table 1:** C4orf3 mRNA expression in ASPC-1 and SUIT-2 cells under normoxic and hypoxic conditions

	Z-Score	Ratio
ASPC-1	3.457	5.261
SUIT-2	4.187	5.758

**Table 2:** Characteristics of the five clinical SCLC cases.

	case1	case2	case3	case4	case5
Age	67	77	75	73	75
Gender	M	M	M	M	M
Tumor location	LLL	LUL	-	RLL	RLL
Surgical procedure	lobectomy	wedge resection	mediastinal lymph node biopsy	wedge resection	wedge resection
Tumor size (mm)	22 x 12	14 x 8	-	25x25	21x12
UICC T category	T1b	T1b	Tx	T1c	T2a
UICC N category	N2	N0	N2	N0	N1
Pleural invasion	PL0	PL0	-	PL0	PL2
Lymphatic permmeation	Ly0	Ly0	-	Ly0	Ly1
Venous invasion	V0	V0	-	V0	V1
Bronchial transection	(-)	(-)	-	(-)	(-)
Stage classification	IIIA	IA2	IIIA	IA3	IIB

M: Male; LLL: Left Lower Lobe; LUL: Light Upper Lobe; RLL: Right Lower Lobe

## Data Availability

The datasets generated and/or analysed in the current study are available from the corresponding author upon reasonable request.

## Abbreviations

SCLC: small cell lung cancer
HIF-1α: hypoxia-inducible factor 1 alpha
C4orf3: chromosome 4 open reading frame 3
PHD: prolyl hydroxylase domain-containing protein
VHL: von Hippel-Lindau tumour suppressor gene product
PIASy: protein inhibitor of activated STAT Y
SUMO: small ubiquitin-related modifier
IHC: immunohistochemistry
