# Supplementary material for: C4orf3 Regulates HIF-1α Degradation Under Hypoxic Conditions and Contributes to the Malignant Phenotype in Small Cell Lung Cancer
Source: J Cancer. 2026 Jan 23;17(3):457–68. doi: 10.7150/jca.127942 (PMC13003554; doi:10.7150/jca.127942)
Supplement: Supplementary file 1 — Supplementary figure. [file jcav17p0457s1.pdf]

Figure S1

C4orf3 expression is upregulated under hypoxic conditions in pancreatic cancer cell lines. (A) Western blotting analysis showing increased C4orf3 protein levels at 0, 24, and 48 h of hypoxic culture.
